# Supplementary material for: Classification of Protein Sequences by a Novel Alignment-Free Method on Bacterial and Virus Families
Source: Genes (Basel). 2022 Sep 27;13(10):1744. doi: 10.3390/genes13101744 (PMC9602327; doi:10.3390/genes13101744)
Supplement: Supplementary file 1 [file genes-13-01744-s001.zip › genes-1912300-supplementary.pdf]

Supplementary Materials: Classification of protein sequences by a novel alignment-free method on bacterial and virus families

Mengcen Guan [1], Leqi Zhao [1] and Stephen S.-T. Yau [1,2,*]

Table S1. The baterial families names in this paper

1. Acetobacteraceae	2. Acidiferrobacteraceae	3. Acidithiobacillaceae
4. Acidobacteriaceae	5. Acuticoccaceae	6. Aeromonadaceae
7. Aestuariivirgaceae	8. Alcaligenaceae	9. Algiphilaceae
10. Alteromonadaceae	11. Anaplasmataceae	12. Ancalomicrobiaceae
13. Aquificaceae	14. Arenicellaceae	15. Aurantimonadaceae
16. Azonexaceae	17. Bartonellaceae	18. Beijerinckiaceae
19. Bradyrhizobiaceae	20. Bryobacteraceae	21. Budviciaceae
22. Burkholderiaceae	23. Caedimonadaceae	24. Caldisericaceae
25. Calditrichaceae	26. Candidatus deianiraeaceae	27. Candidatus magnetaquicoccaceae
28. Candidatus midichloriaceae	29. Candidatus paracaedibacteraceae	30. Cardiobacteriaceae
31. Caulobacteraceae	32. Cellvibrionaceae	33. Chelatococcaceae
34. Chromatiaceae	35. Chromobacteriaceae	36. Chrysiogenaceae
37. Cohaesibacteraceae	38. Colwelliaceae	39. Coprothermobacteraceae
40. Coxiellaceae	41. Crenotrichaceae	42. Deferribacteraceae
43. Desulfurobacteriaceae	44. Dictyoglomaceae	45. Ectothiorhodospiraceae
46. Elioraeaceae	47. Elusimicrobiaceae	48. Emcibacteraceae
49. Endomicrobiaceae	50. Enterobacteriaceae	51. Erwiniaceae
52. Ferrimonadaceae	53. Ferrovaceae	54. Fusobacteriaceae
55. Gallionellaceae	56. Geminicoccacea	57. Granulosicoccaceae
58. Hafniaceae	59. Halieaceae	60. Holophagaceae
61. Holosporaceae	62. Hydrogenothermaceae	63. Hyphomicrobiaceae
64. Hyphomonadaceae	65. Idiomarinaceae	66. Immundisolibacteraceae
67. Kiloniellaceae	68. Kordiimonadaceae	69. Legionellaceae
70. Leptotrichiaceae	71. Magnetococcaceae	72. Methylobacteriaceae
73. Methylocystaceae	74. Methylophilaceae	75. Methylothermaceae
76. Microbullbiferaceae	77. Minwuiaceae	78. Morganellaceae
79. Moritellaceae	80. Neisseriaceae	81. Nitrosomonadaceae
82. Nitrospinaceae	83. Nitrospiraceae	84. Notoacmeibacteraceae
85. Parvularculaceae	86. Pectobacteriaceae	87. Pelagibacteraceae
88. Phyllobacteriaceae	89. Porticoccaceae	90. Pseudoalteromonadaceae
91. Psychromonadaceae	92. Pyrinomonadaceae	93. Rhizobiaceae
94. Rhodobiaceae	95. Rhodocyclaceae	96. Rhodothalassiaceae
97. Rickettsiaceae	98. Roseiarcaceae	99. Salinarimonadaceae
100. Shewanellaceae	101. Sinobacteraceae	102. Sneathiellaceae
103. Sphingomonadaceae	104. Spongiibacteraceae	105. Steroidobacteraceae
106. Sterolibacteriaceae	107. Succinivibrionaceae	108. Sutterellaceae
109. Thermithiobacillaceae	110. Thermoanaerobaculaceae	111. Thioalkalibacteraceae
112. Thioalkalispiraceae	113. Thorselliaceae	114. Vicinamibacteraceae
115. Wenzhouxiangellaceae	116. Yersiniaceae	117. Zoogloeaceae

Table S2. The classification result of *Mycobacteriaceae* dataset

Enzyme class	Total number	Correct number	Accuracy
Oxidoreductases	958	921	0.961378
Transferases	2538	2471	0.973601
Hydrolases	1383	1309	0.946493
Lyases	705	689	0.977305
Isomerases	299	281	0.939799
Ligases	743	726	0.977120
Translocases	264	255	0.965909
Total	6890	6652	0.965457

Table S3. The classification result of *Xanthomonadaceae* dataset

Enzyme class	Total number	Correct number	Accuracy
Oxidoreductases	482	470	0.975104
Transferases	1478	1460	0.987821
Hydrolases	757	731	0.965654
Lyases	414	405	0.978261
Isomerases	251	246	0.980080
Ligases	480	478	0.995833
Translocases	155	150	0.967742
Total	4017	3940	0.980831

Table S4. The classification result of *Vibrionaceae* dataset

Enzyme class	Total number	Correct number	Accuracy
Oxidoreductases	528	499	0.945076
Transferases	1717	1685	0.981363
Hydrolases	903	863	0.955703
Lyases	483	466	0.964803
Isomerases	284	268	0.943662
Ligases	494	488	0.987854
Translocases	256	242	0.945313
Total	4665	4511	0.966989

Table S5. The virus families names and their number of protein sequences in this paper

Virus family	Number of protein sequences	Number of protein sequences with missing information
Adenoviridae	418	0
Alloherpesviridae	76	0
Alphaflexiviridae	97	0
Alphatetraviridae	5	0
Ampullaviridae	53	0
Anelloviridae	72	0
Arteriviridae	40	1
Ascoviridae	34	0
Astroviridae	32	0
Bacilladnaviridae	8	0
Baculoviridae	407	0
Benyviridae	12	0
Betaflexiviridae	58	0
Bicaudaviridae	72	0
Birnaviridae	35	0
Botourmiaviridae	3	0
Bromoviridae	109	0
Caliciviridae	57	0
Caulimoviridae	80	0
Chrysoviridae	4	0
Circoviridae	27	17
Closteroviridae	31	0
Coronaviridae	468	0
Cystoviridae	13	0
Dicistroviridae	6	0
Flaviviridae	130	4
Fuselloviridae	35	0
Geminiviridae	198	22
Haploviricotina	839	0
Hepadnaviridae	295	0
Hepeviridae	31	0
Herelleviridae	51	0
Herpesviridae	2082	4
Hypoviridae	4	0
Inoviridae	112	0
Iridoviridae	449	0
Kitaviridae	6	0
Lavidaviridae	21	0
Leviviridae	33	0
Lipothrixviridae	165	0
Luteoviridae	53	0
Microviridae	80	0
Mimiviridae	909	0
Myoviridae	606	10
Nanoviridae	51	0
Narnaviridae	3	0
Nodaviridae	23	0
Papillomaviridae	581	0
Partitiviridae	6	0
Parvoviridae	76	0
Phycodnaviridae	29	0
Picobirnaviridae	3	0
Picornaviridae	99	7
Pleolipoviridae	9	0
Podoviridae	372	0
Polydnaviridae	53	0
Polyomaviridae	91	0
Polyploviricotina	1807	0
Potyviridae	86	0
Poxviridae	1376	6
Reoviridae	806	2
Retroviridae	1042	7
Rudiviridae	43	0
Secoviridae	57	0
Siphoviridae	539	2
Solemoviridae	16	0
Tectiviridae	26	0
Tobaniviridae	20	0
Togaviridae	62	3
Tombusviridae	80	0
Totiviridae	13	0
Tymoviridae	29	0
Virgaviridae	132	3

Table S6. 11 intersecting dsDNA virus family pairs in 250-dimension space

Podoviridae	Siphoviridae
Iridoviridae	Siphoviridae
Myoviridae	Siphoviridae
Herpesviridae	Iridoviridae
Herpesviridae	Poxviridae
Herpesviridae	Reoviridae
Myoviridae	Podoviridae
Iridoviridae	Mimiviridae
Iridoviridae	Poxviridae
Iridoviridae	Myoviridae
Mimiviridae	Poxviridae

Table S7. The results of convex hull intersection in 2-dimension space based on 250-dimensional accumulated natural vector: Percentage of non-intersection is the percentage of disjoint convex hull pairs of all convex hull pairs under one virus family

Virus family	Percentage of non-intersection	Virus family	Percentage of non-intersection
Adenoviridae	0.6667	Hepeviridae	0.9861
Alloherpesviridae	0.8472	Herelleviridae	0.8472
Alphaflexiviridae	0.8056	Herpesviridae	0.1389
Alphatetraviridae	1.0000	Hypoviridae	1.0000
Ampullaviridae	0.8472	Inoviridae	0.8056
Anelloviridae	0.9861	Iridoviridae	0.5333
Arteriviridae	0.9583	Kitaviridae	0.9861
Ascoviridae	0.9444	Lavidaviridae	0.9583
Astroviridae	1.0000	Leviviridae	0.9444
Bacilladnaviridae	1.0000	Lipothrixviridae	0.8194
Baculoviridae	0.6389	Luteoviridae	0.9167
Benyviridae	0.9861	Microviridae	0.8194
Betaflexiviridae	0.875	Mimiviridae	0.375
Bicaudaviridae	0.8611	Myoviridae	0.5278
Birnaviridae	0.9861	Nanoviridae	0.9306
Botourmiaviridae	1.0000	Narnaviridae	1.0000
Bromoviridae	0.9444	Nodaviridae	0.9861
Caliciviridae	0.9861	Papillomaviridae	0.7083
Caulimoviridae	0.8889	Partitiviridae	0.9861
Chrysoviridae	1.0000	Parvoviridae	0.8056
Circoviridae	0.9028	Phycodnaviridae	0.9306
Closteroviridae	0.9306	Picobirnaviridae	1.0000
Coronaviridae	0.8056	Picornaviridae	0.875
Cystoviridae	0.9583	Pleolipoviridae	0.9861
Dicistroviridae	1.0000	Podoviridae	0.6806
Flaviviridae	0.9861	Polydnaviridae	0.8472
Fuselloviridae	0.8889	Polyomaviridae	0.8333
Geminiviridae	0.8333	Polyploviricotina	0.7083
Haploviricotina	0.4583	Potyviridae	0.9583
Hepadnaviridae	0.9583	Poxviridae	0.3611
Reoviridae	0.5694	Tobaniviridae	0.9861
Retroviridae	0.5000	Togaviridae	1.0000
Rudiviridae	0.9028	Tombusviridae	0.8889
Secoviridae	0.9861	Totiviridae	0.9861
Siphoviridae	0.5278	Tymoviridae	0.9861
Solemoviridae	0.9861	Virgaviridae	0.8611
Tectiviridae	0.9028		

Table S8. The results of convex hull intersection in 2-dimension space based on 60-dimensional natural vector: Percentage of non-intersection is the percentage of disjoint convex hull pairs of all convex hull pairs under one virus family. All convex hulls intersect in 2-dimension space based on 60-dimensional natural vector. So some families are omitted.

Virus family	Percentage of non-intersection	Virus family	Percentage of non-intersection
Adenoviridae	0.0000	Hepeviridae	0.0000
Alloherpesviridae	0.0000	Herelleviridae	0.0000
Siphoviridae	0.0000	Tymoviridae	0.0000
Solemoviridae	0.0000	Virgaviridae	0.0000
Tectiviridae	0.0000		
